# The microRNA miR-14 Regulates Egg-Laying by Targeting EcR in Honeybees (*Apis mellifera*)

**DOI:** 10.3390/insects12040351

**Published:** 2021-04-14

**Authors:** Xiao Chen, Jinluan Fu

**Affiliations:** 1 Institute of Apicultural Research, Chinese Academy of Agricultural Sciences, Beijing 100093, China; 2 National Engineering Laboratory for Animal Breeding & Key Laboratory of Animal Genetics, Breeding and Reproduction, College of Animal Sciences and Technology, China Agricultural University, Beijing 100193, China; fujinlian@126.com

**Keywords:** honeybees, egg number, miRNA, EcR

## Abstract

**Simple Summary:**

Honeybees (Apis mellifera) are important pollinators and commonly used for honey production. The oviposition behavior in honeybees is complex and errors in oviposition could affect the development of the bee colony. Ecdysone receptor (EcR) and miR-14 were previously reported to play important roles in egg-laying. Moreover, EcR was predicted to be the target gene of miR-14 and may form miR-14-EcR cross-talk. By knocking down and overexpression of miR-14 and EcR in queen model, the effect of RNA expression of miR-14 and EcR on the number of eggs laid by honeybee queens was analyzed. We found that the expression of miR-14 and EcR was associated with the egg number of queens. In specific, inhibition of miR-14 expression enhanced the egg number, while overexpression of EcR enhanced the egg number in honeybee queens.

**Abstract:**

Honeybees (*Apis mellifera*) are important pollinators and are commonly used for honey production. The oviposition behavior in honeybees is complex and errors in oviposition could affect the development of the bee colony. Recent studies reported that RNA–RNA cross-talk played a critical role in several biological processes, including reproduction. Ecdysone receptor (EcR) and miR-14 were previously reported to play important roles in egg-laying. Moreover, EcR was predicted to be the target gene of miR-14 and may form miR-14-EcR cross-talk. In this study, knocking down and overexpression of miR-14 and EcR in queen model were implemented. The effect of RNA expression of miR-14 and EcR on the number of eggs laid by honeybee queens were analyzed. Further, luciferase assay was used to confirm the target relation between miR-14 and 3′UTR of EcR. The results showed that the expression of miR-14 and EcR was associated with the number of eggs laid by queens. In specific, inhibition of miR-14 expression enhanced the number of eggs laid, while overexpression of EcR enhanced the number of eggs laid. Lastly, we determined that miR-14 directly targets the mRNA of EcR. These findings suggest that the cross-talk of miR-14-EcR plays an important role in the number of eggs laid by honeybee queens.

## 1. Introduction

Honeybees (*Apis mellifera*) are important pollinators and are commonly used for honey production [1]. Among the main traits of honeybees, oviposition behavior is an important one. The oviposition behavior in honey bees is complex and errors in oviposition could affect the development of the bee colony [2]. However, most reproductive traits are complex in terms of their genetic architecture, present low heritability and are sex-limited [3,4]. Thus, it is hard to be improved by using traditional selection methods, e.g., selective breeding. With the development of molecular technologies, new approaches are being applied to improve reproductive traits and other complex traits, such as marker-assisted selection (MAS) and genomic selection [5,6]. These methods have been used widely in domestic animals for years. However, in honeybees, these strategies became popular only in recent years [6].

One manifestation of the oviposition behavior is the number of eggs laid by queens. Honeybees provide an excellent model for egg-laying studies, since queens specialize in egg-laying. The queens are equipped with huge ovaries and with an egg-laying rate of approximately 2000 eggs per day for several years under ideal circumstances [7]. Numerous studies have focused on the molecular mechanisms underlying the genetic mechanisms of egg-laying of honeybee queens. Most studies have focused on gene expression profiles to identify factors that control and regulate queens’ fertility. Comparisons have been performed in various biological contexts ranging from gene-by-gene analyses [8,9,10] to large-scale genetic studies [11,12,13,14]. In recent years, studies also found that non-coding RNAs, such as microRNAs (miRNAs), lncRNAs, and circRNAs, play roles in a potentially complex network that regulates egg-laying. Several candidate genes and miRNAs are reported to play important roles in egg-laying of queens. For example, ecdysone receptor (EcR), mushroom body large-type Kenyon cell-specific protein-1 (MBLK-1), ecdysone-induced protein 74 (E74) and ultraspiracle (Usp) [15]. These genes are ecdysteroid hormones (Ec, used hereafter to refer to all types of ecdysteroids) response genes and participate in Ec signaling. Ec are the major steroid hormones of insects best known for their role in several crucial biological processes, including egg-laying [16,17,18]. In addition, several miRNAs, such as bantam, miR-8, miR-14, miR-184, and miR-315, have been reported to play important roles in ovary development and caste determination in honeybees [7,19]. Additionally, miR-14 has been suggested to be associated with juvenile hormones (JH) and ecdysteroids (Ec), which play key roles in ovary development and other reproductive behaviors in honeybees [20,21,22,23,24,25,26].

A better understanding of the genetic architecture of honeybees will help scientists develop a better strategy for acceleration of the genetic improvement of the reproductive traits. Our previous study found that differentially expressed coding and non-coding RNAs in ovary have effect on ovary development and oviposition in honeybee queens. In this study, we focus on miR-14 and EcR. Our previous study found that in the egg-laying initiation process, the expression of miR-14 down-regulated significantly (*p* < 0.01), and EcR expression up-regulated significantly (*p* < 0.01) [27]. We also found EcR had binding site of miR-14, and they form EcR-miR-14 cross-talk [27]. In Drosophila, EcR’s expression and activity levels have been shown to be negatively regulated by miR-14 [28]. In honeybees, miR-14 was found to be down-regulated in Ec knock down bees, which indicated it was an Ec response genes. EcR was also an Ec response gene [20,21,23,29]. Ec have been proved to affect egg-laying [15,17,29]. miR-14 and EcR may interact with each other and play roles in egg-laying by participating in Ec signaling. However, it was not clear whether the RNA expression of miR-14 and EcR was directly associated with the number of eggs laid by queens, and how the expression of miR-14 and EcR were related during honeybees’ egg-laying. In the present study, we further investigate the role of miR-14 and EcR in egg-laying of honeybees. The knocking-down and overexpression of miR-14 and EcR in queen model were implemented. The effect of RNA expression of miR-14 and EcR on the number of eggs laid was analyzed. Further, luciferase assay was used to confirm the target relation between miR-14 and 3′UTR of EcR. We show that the decrease of miR-14 expression and increase of EcR expression have a positive effect on the number of eggs laid in honeybee queens. Furthermore, we found that miR-14 directly targets the mRNA of EcR.

## 2. Materials and Methods

### 2.1. Ethics Statement

The apiaries for honeybee sample collection were maintained by Institute of Apicultural Research, Chinese Academy of Agricultural Sciences (IAR, CAAS), Beijing, China. The sample collection was approved by the ethics committee of IAR, CAAS. Honeybees were humanely sacrificed as necessary to ameliorate suffering. No specific permits were required for the described studies.

### 2.2. Sampling

All samples were obtained from *Apis mellifera ligustica* honeybee colonies. In June 2020, 100 sister queens from a single source colony were reared using standard beekeeping techniques [30]. Five days before the emergence, the queens were transferred to an incubator at 36 °C and kept individually in plastic vials. One day old, the queens were marked and each was introduced to her own nucleus colony. The strength of each colony was similar. The entrance of each hive was covered with a queen excluder that confined the queen within the hive but allowed workers to exit and enter.

Six-day-old queens were implemented instrumental insemination (QueenBee Artificial Insemination Instrument VCFE-QBAII-H1.3, Victory & Explore Ltd., Shanghai, China). For instrumental insemination, the source and quantity of the semen were the same for all mated queens. The source and quantity of the semen was the same for all mated queens. For semen collection, around 1000 drones were used from three different queens’ families with different genetic background. For insemination, 8μL mixed sperm was used for each queen. Queens that successfully laid eggs were included in the RNA injection experiment. For each queen, around 800 eggs that were laid by the treated queen were examined a day before RNA injection.

Injection of queens was implemented according to Amdam’s methods [31]. Queens were injected with 1 μL dsRNA solution (5 μg/μL) or 1 μL nonsense sequence. Injections were made dorsally between the 5th and 6th abdominal segments. The queens stayed in the fixed position for one hour before introduction into the original colony. Eggs were examined the next day after injection. The number of eggs per day was recorded using Population Measurment Liebefeld method for three days (Figure S1). After the third time of recording the number of eggs, queens were collected. Each sample consisted of a single queen. Ten samples per treatment group were used for RNA extraction (total = 90 samples).

### 2.3. Oversupply/Inhibition of miR-14 and EcR in Honeybee Queens

Ago-mir14 with the sense strand (sense: 5′UCAGUCUUUUUCUCUCUCCUA3′, antisense: 5′GGAGAGAGAAAAAGACUGAUU3′) and the stable nonsense control strand (sense: 5′ UUCUCCGAACGUGUCACGUTT3′, antisense: 5′ACGUGACACGUUCGGAGAATT3′) synthesized by GenPharma (Shanghai, China). An inhibitor, antago-mir14 (5′ UAGGAGAGAGAAAAAGACUGA3′) and inhibitor nonsense control (5′CAGUACUUUUGUGUAGUACAA3′) was also synthesized. For EcR overexpressed vector, EcR cds fragment (1890-bp, NM_001098215.2, cds sequence was the same for EcR-A and EcR-B) was synthesized and amplified using 2 x SG PCRMasterMix (SinoGene, China). The EcR cds was cloned into a pCDNA3.1 vector. EcR siRNA (siRNA1: 5′-GATCCTTACAGTCCCAACAGT-3′, siRNA2: 5′-GAACGCGGTCTATCAGTGTAA-3′; siRNA3: 5′-GAGATGTGCTTCAGCCTCAAGT-3′), and siRNA nonsense control (sense: 5′-UUCUCCGAACGUGUCACGUTT-3′) was also synthesized. Injection with siRNA or nonsense sequence, and sampling queens were implemented as described in 2.2.

### 2.4. RNA Isolation and Real Time Quantitative PCR

Total RNA was extracted from samples using Trizol reagent (Invitrogen, Carlsbad, CA, USA) according to the manufacturer’s instructions. The purity of RNA was checked using the NanoPhotometer spectrophotometer (IMPLEN, Westlake Village, CA, USA), and the concentration was measured using Qubit RNA Assay Kit in Qubit 2.0 Flurometer (Life Technologies, Carlsbad, CA, USA). The integrity of RNA was assessed using the RNA Nano 600Assay Kit of the Agilent Bioanalyzer 2100 system (Agilent Technologies, Santa Clara, CA, USA).

For mRNA amplification, for each sample, first strand cDNA was synthesized using 1 μg of total RNA. M-MLV FIRST STRAND KIT (Invitrogen, Shanghai, China) and oligo (dT)18 primer were used in a total of 20 μL reverse transcription reaction following the supplier’s instruction. Transcript-specific primer pairs (Table 1) were designed with Oligo 6.0 software. Standard PCRs on cDNA were carried out to verify amplification sizes. Transcript quantification was performed using SYBR Green mix (Roche Diagnostics GmbH, Roche Applied Science, Mannheim, Germany) in a Roche LightCylcer 480 (Roche Diagnostics GmbH, Roche Applied Science, Mannheim, Germany). The RT-PCR reactions were prepared in a total volume of 20 μL containing 5 μL of cDNA (50 ng, 1:100 dilution), 10 μL of SYBR Green mix, 3 μL water contained in the kit and 0.02 μmol/L of both forward and reverse gene-specific primers. β-actin served as the internal reference gene. Cycling conditions were 95 °C for 10 min, followed by 45 cycles of 95 °C (10 s) and 60 °C (10 s) where the fluorescence was acquired. Finally, a dissociation curve to test PCR specificity was generated from one cycle at 95 °C (10 s) followed by 60 °C (1 min) and ramp up to 95 °C with acquired fluorescence during the ramp to 0.2 °C/s.

For miRNA amplification, for each sample, first strand cDNA was synthesized using 1 μg of total RNA. miRcute Plus miRNA First-strand cDNA Kit (TIANGEN, Beijing, China) was used in a total of 20 μL reverse transcription reaction following the supplier’s instructions. Transcript quantification was performed using miRcute Plus miRNA qPCR Kit (SYBR Green) in a Roche LightCylcer 480 (Roche Diagnostics GmbH, Roche Applied Science, Mannheim, Germany). The RT-PCR reactions were prepared in a total volume of 20 μL containing 5 μL of cDNA (50 ng, 1:100 dilution), 10 μL of miRcute Plus miRNA PreMix, 10 μM Reverse primer contained in the kit and 10 μM forward primer (see Table 1). U6 served as the internal reference gene. Cycling conditions were 95 °C for 15 min, followed by 45 cycles of 94 °C (20 s) and 60 °C (34 s) where the fluorescence was acquired.

PCR efficiency of each gene was estimated by standard curve calculation using four points of cDNA serial dilutions. Ct values were transformed to quantities using the comparative Ct method. Relative gene expression was calculated using the 2^−ΔΔCt^ method. Comparisons of gene expression levels were made using a t-test.

### 2.5. S2 Cell Culture, Luciferase Reporter Assay and Western-Blot

A 575-bp fragment from EcR 3′ UTR and its mutant sequence were synthesized and amplified using 2 × SG PCRMasterMix (SinoGene, China) (Table S1). The EcR 3′ UTR and its mutant were cloned into a psiCHECK2 vector, respectively. For miR-14, agomir and antagomir were used, respectively. Drosophila S2 cells were cultured with 10% fetal bovine serum (HyClone) in Schneider’s Insect Medium (Invitrogen, Carlsbad, CA, USA). Cells were seeded at 106 cells per well in a 12-well plate. One day later, miR-14 mimics was co-transfected with either psiCHECK2- EcR 3′UTR, psiCHECK2- EcR 3′UTR-m, or an empty vector in the cells using the calcium phosphate transfection method, as described by Tiscornia et al. [32]. Twenty-four and forty-eight hours after transfection, luciferase assays were performed using a dual-specific luciferase assay kit (Biyuntian, Shanghai, China). Renilla luciferase activity provided normalization for firefly luciferase activity.

To confirm the regulation mechanism of miR-14 on EcR, miR-14 agomir and antagomir were co-transferred with EcR overexpressed vector. For the EcR overexpressed vector, we sub-cloned the cds fragment of EcR mRNA, which was the homologous sequence of EcR-A and EcR-B, into a luciferase reporter plasmid designated as psiCHECK2- EcR. Twenty-four and forty-eight hours after transfection, the mRNA expression of EcR was detected using qPCR, and protein of EcR was detected using Western blot. Methods of mRNA extraction and qPCR amplification were described in 2.4. For Western blot, proteins were extracted from S2 cells using the Cell Total Protein Extraction Kit (Sangon Biotech, Shanghai, China). The protein samples were separated through a 4% denaturing polyacrylamide gel, and transferred to nitrocellulose membranes (Pall Life Sciences, Shanghai, China). Non-specific binding-sites on the membranes were blocked with 5% nonfat milk in TBST for 1.5 h at room temperature. The membrane was incubated with TBST containing 5% nonfat milk and diluted rabbit anti-EcR polyclonal antibody (1: 500) (made by ourselves, peptide sequence: LVLPSGVNMC) overnight at 4 °C. It was then washed, incubated with horseradish peroxidase-labeled anti-rabbit IgG (1: 3000) (AC028, Abclonal, Wuhan, China) for an hour at room temperature, and washed again. Finally, the membrane was colored using the DAB kit (Invitrogen, Carlsbad, CA, USA) and exposed using Chemiluminescence Detection Kit for HRP (Sunbio, Shanghai, China). Scanned images were quantified using Image J analysis software.

## 3. Results

### 3.1. miR-14 Expression and Its Effect on Egg Number of Queens

**Figure 1:**
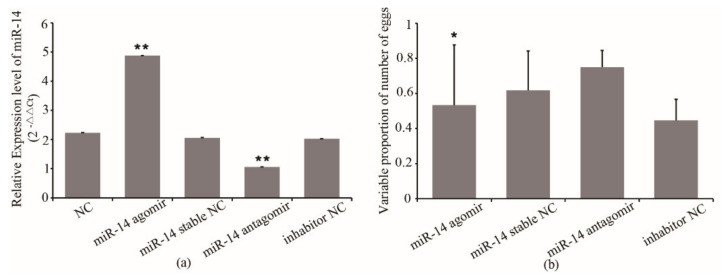
(**a**) The expression of miR-14 in queens after treatment and (**b**) the effect of its expression on the egg number of queens. NC, queens without treatment. miR-14 stable NC, miR-14 stable nonsense sequence control. Inhibitor NC, inhibitor nonsense sequence control. * means *p* < 0.05, ** means *p* < 0.01.

Considering the importance of miR-14 in oviposition of queens, we decided to overexpress and inhibit its expression to examine the possible effects on the number of eggs laid by queens. The miR-14 antagomir and agomir were injected into queens. The qRT-PCR confirmed the overexpression and inhibition of miR-14 in queens, respectively. As shown in Figure 1, the miR-14 expression in queens from the agomir group increased by 137% compared with that of the agomir stable NC group, while miR-14 expression from the antagomir group decreased by 48% compared with that of the inhibitor NC group (Figure 1a).

To investigate the possible effect of miR-14 on the egg number in queens, the association between the miR-14 expression and the egg number of queens was analyzed. The results showed that the egg number of queens from the miR-14 agomir group decreased by 35% compared with that of the agomir stable NC group (*p* < 0.05) (Figure 1b). The egg number of queens from the miR-14 antagomir group increased by 50% compared with that of the inhibitor NC group (Figure 1b). However, the egg number of queens from the agomir stable NC and the inhibitor NC group was only 72~74% that of their original level (Figure 1b). The results indicated that the expression of miR-14 has an effect on the egg number of queens. Inhibition of miR-14 expression improved the egg number. However, the damage caused by the injection to queens may also affect the egg number.

### 3.2. EcR Expression and Its Effect on the Egg number of Queens

**Figure 2:**
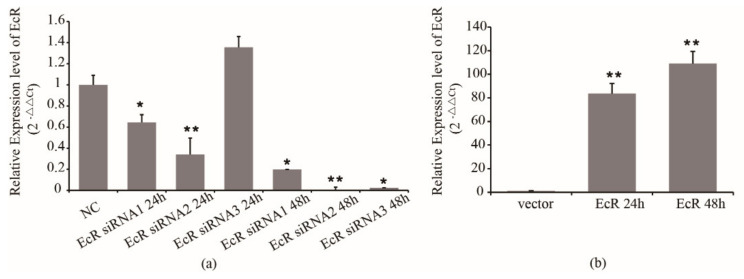
Effect of siRNAs and overexpressed vector on the expression of EcR. (**a**) Effect of siRNA on the expression of EcR. (**b**) Effect of overexpressed vector on the expression of EcR. * means *p* < 0.05, ** means *p* < 0.01.

Firstly, we detected the efficiency of siRNA in S2 cells. We transfected EcR siRNA1, siRNA2, siRNA3 and NC into S2 cells, and used RT-PCR to detect the mRNA expression of EcR. The results showed that the inhibition caused by siRNA1, siRNA2, and siRNA3 was effective (Figure 2a). In the next steps, siRNA2 was used to inhibit the EcR expression.

On the other hand, we subcloned the cds fragment of EcR mRNA, which is a homologous sequence of EcR-A and EcR-B, into a luciferase reporter plasmid named psiCHECK2-EcR. We transfected blank vector and psiCHECK2-EcR into S2 cells, respectively. Then, cells were collected at 24 h and 48 h after transfection to detect the expression of EcR. The results showed that after transfection, the expression of EcR increased significantly at 24 h, and was increased around 100 times at 48 h (Figure 2b).

**Figure 3:**
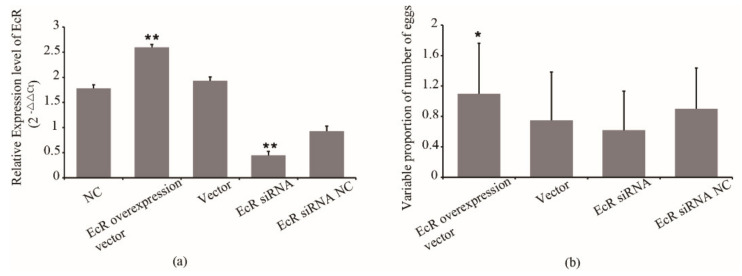
(**a**) The expression of EcR in queens after treatment and (**b**) the effect of its expression on the egg number of queens. EcR siRNA NC, EcR siRNA nonsense sequence control. Normal control, queens without treatment. * means *p* < 0.05, ** means *p* < 0.01.

EcR was also reported to relate with the oviposition of queens. We overexpressed and inhibited the expression of EcR to examine possible effects on the egg number of queens. The synthetic siRNA and overexpression vector were injected into queens. The qRT-PCR confirmed the overexpression and inhibition of EcR in queens, respectively. As shown in Figure 3a, the EcR expression in queens from the EcR overexpression group increased by 34% compared with that of the blank vector group, while EcR expression from the siRNA group decreased by 52% compared with that of the siRNA NC group. The association between the EcR expression and the egg number of queens was also analyzed. The results showed that the egg number of queens from the EcR overexpressed group increased by 94% compared with that of the blank vector group (*p* < 0.05) (Figure 3b). The egg number of queens from the siRNA group decreased by 31% compared with that of the inhibitor NC group (Figure 3b). However, the egg number of queens from the blank vector group was only 75% that of its original level and the egg number of queens in the inhibitor NC group was 90% that of its original level (Figure 3b). The results indicated that the expression of EcR has an effect on the egg number of queens. Overexpression of EcR improved the egg number.

### 3.3. Confirmation of the Interaction of miR-14 with EcR Using a Luciferase Reporter Assay

**Figure 4:**
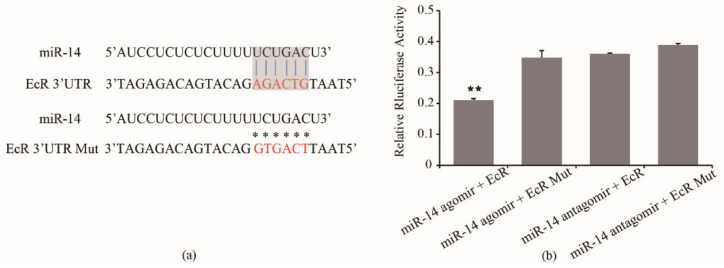
(**a**) Sequences of the interaction sites between miR-14 and EcR 3′ UTR and (**b**) co-transfection of psiCHECK2- EcR 3′ UTR resulted in dramatic suppression of the luciferase activity. a. Blue line indicates interaction sites, and asterisks indicate mutated site. Nucleotides of interaction sites and mutated sites are shown in red. Grey shaded areas indicate canonical 7mer “seed” region that aligns with the target site, the vertical lines indicate contiguous Watson–Crick pairing. b. A normalized firefly/renilla luciferase value was plotted with ±SD. ** means *p* < 0.01.

To test whether miR-14 actually targets the EcR 3′ UTR, we sub-cloned a 517-bp fragment of the 3′ UTR region of EcR mRNA that included the predicted miR-14 recognition site (Figure 4a) into a luciferase reporter plasmid designated as psiCHECK2-EcR 3′UTR (short for W). A sequence with mutations (m) was also constructed as the negative control for the same reporter assay, named psiCHECK2-EcR 3′UTR-m (short for M). miR-14 mimics and inhibitor were directly used for co-transferred. When miR-14 mimics was co-transfected with psiCHECK2-EcR 3′UTR in S2 cells, the luciferase activity significantly decreased compared to the assay involving co-transfection with psiCHECK2- EcR 3′UTR-m (*p* < 0.01, Figure 4b). The results indicated that miR-14 targeted the EcR 3′UTR.

### 3.4. The Regulation Mechanism of miR-14 on EcR

**Figure 5:**
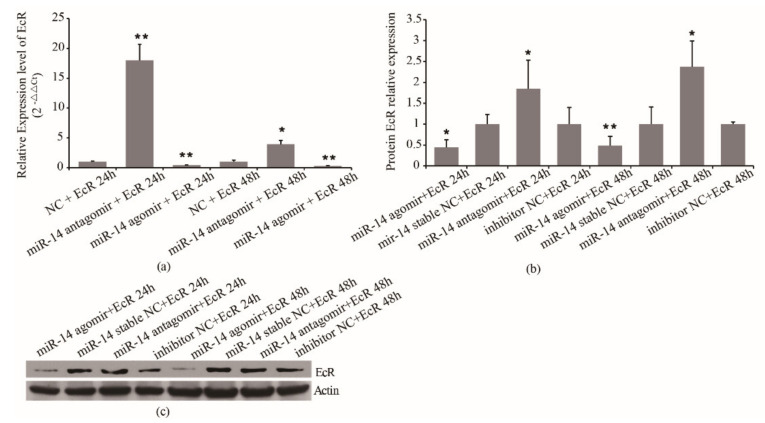
Effect of miR-14 on mRNA and protein level of EcR. (**a**) Effect of miR-14 on mRNA level of EcR. (**b**) Western blot result. (**c**) Effect of miR-14 on protein level of EcR at 24 h and 48 h after treatment. * denoting *p* < 0.05, ** denoting *p* < 0.01. Data are from three replicates. β-actin was used as the reference protein.

To further confirm the regulation mechanism of miR-14 on EcR, miR-14 agomir/antagomir and psiCHECK2- EcR were co-transfected in S2 cells. The mRNA expression of miR-14 and EcR was detected by RT-PCR, and the expression of EcR protein was detected by Western blot. The results showed that in the miR-14 inhibition group, the expression of miR-14 decreased by 70% at 24 h and 50% at 48 h, respectively (Figure 5a). In the miR-14 overexpressed group, the expression of miR-14 increased by 145% at 24 h and 141% at 48 h, respectively (Figure 5a). For the expression of EcR, in the miR-14 inhibition group, the mRNA expression of EcR increased almost 18 times at 24 h and increased around 3.9 times at 48 h, respectively (Figure 5b). In the miR-14 overexpressed group, the mRNA expression of EcR decreased by 60% at 24 h and 70% at 48 h, respectively (Figure 5b). Western blot results showed that in the miR-14 overexpressed group, the protein expression of EcR decreased by 56% at 24 h and 51% at 48 h, respectively (Figure 5c). In the miR-14 inhibition group, the protein expression of EcR increased by 85% at 24 h and 138% at 48 h, respectively (Figure 5c). The results showed that miR-14 affects EcR protein expression by regulating the expression of EcR mRNA. It indicated that miR-14 may inhibit the transcription of EcR mRNA.

## 4. Discussion

Ec is recognized as one of the most important hormones regulating the reproductive activities of honeybees [16,17,33]. Studies showed that the Ec titer in the ovaries of queens and workers with outstanding reproductive ability was significantly high [12,15]. During the first time of oviposition, Ec titer in queens’ ovary increased significantly [16,17,18]. In *Bumble terrestris*, large amounts of Ec are produced in the ovaries of queens and reproductively dominant workers, which are reflected by high Ec titers in the hemolymph of these bees [16,17]. The dynamic regulation of Ec in adult honeybees, and the growing evidence that many behavioral changes in honeybees are associated with differential expression of Ec responsive genes [15]. EcR is one of the most important Ec responsive genes. EcR is the receptor of Ec, and Ec and EcR combined to form Ec response elements (EcRE) regulating the Ec titer [12], and further affecting the oviposition of honeybees [16,17,18,19]. The key role of EcR for Ec signaling in honeybees was demonstrated by RNAi-mediated down-regulation of both EcR transcripts (A and B) during pharate-adult development. EcR down-regulation caused differential expression of 234 transcripts, and also promoted the differential expression of 70 miRNAs [4]. This suggested an additional tier of regulation that is mediated by the action of miRNAs on gene expression [4]. In recent years, the role of miRNA in reproduction has been well established. Several miRNAs were found involved in the ovarian functional remodeling and ovarian development in honeybees [7,19]. Researchers also found that miRNAs were related to Ec and EcR [4,27,34]. Especially, miR-14 is found closely related to the activation state of honeybee ovaries and it was reported to involve in regulating the expression and activity of EcR [4,7]. What is important, the gene sequence of EcR has miR-14 binding site [4]. Further, our previous study found that the expression of miR-14 was significantly down-regulated in egg-laying queens than that of virgins [27]. Therefore, our hypothesis is miR-14 affects egg-laying by targeting EcR [17,35,36,37]. We deduced that there is a good correlation between miR-14 and honeybees’ reproductive changes.

However, it was not clear whether the expression of miR-14 and EcR was directly associated with egg numbers of queens, and the mechanism of mir-14 regulating the EcR’s expression is also not clear. In this study, we concentrated on miR-14 and EcR. We recorded the number of eggs per day of each queen by using Population Measurment Liebefeld method, a method regularly used to accomplish this. We detected that the expression of EcR and mir-14 was associated with the number of queens. In the group of EcR overexpression, the egg number was significantly higher than that of the control group. This was consistent with the result in bumblebees, which showed that EcR promoted oviposition [17]. In the group of miR-14 inhibition, the number of eggs was significantly higher than that of the miR-14 stable control group. The number of eggs in the group of miR-14 overexpression was decreased compared with inhibitor control group. Inhibition of miR-14 enhanced the number of eggs (*p* < 0.05). These results are consistent with other species, in which miRNAs was shown to a play role in oocyte maturation [38]. In addition, the expression of miR-14 is largely confined to the ovaries of honeybee queens [27]. Thus, we deduced that there is a good correlation between the expression of EcR and miR-14 and honeybees’ egg number.

We have previously predicted EcR to be a possible target for miR-14 [27]. Our luciferase assay confirmed that miR-14 targets the 3′UTR of EcR because transfection of psiCHECK2-EcR 3′UTR reduced the luciferase activity and psiCHECK2-EcR 3′UTR m rescued this suppression to the same level as that of the blank control (Figure 4). Moreover, we detected the expression of EcR in groups of miR-14 inhibition/overexpression. The result showed that overexpression of miR-14 significantly inhibited the mRNA and protein expression of EcR in queens. These results strongly indicate that miR-14 directly targets EcR. Combining results of the association between the egg number and gene expression, we deduced that miR-14 may be involved in the regulation of egg-laying of honeybees through its target gene EcR.

In the technical aspect, the damage caused by the injection to queens may also affect the number of eggs laid since the number of eggs laid decreased slightly in the NC group. In previous studies, the siRNA injection was usually implemented in pupae or adult workers [4,39]. The observed behaviors were usually growth and development, nursing and foraging [4,39]. These observed behaviors probably were not sensitive to the injection damage. However, the queens may be sensitive to the damage by the injection. The number of eggs laid by queens may be one of the easily observed reflect. The thinner syringe needle may reduce the injection damage. Further studies are needed to confirm it.

The number of eggs laid by honeybee queens was not determined by one singular cause. The number of eggs laid was one of the most important aspects of fecundity in honeybees. Among the factors that influence the number of eggs laid, Ec was one of the most important factors. It is reported that high Ec titer in queens’ ovaries improves the number of eggs laid. Egg-laying, which is hormonally controlled, is affected by many genes that have various cross-talks with other coding or non-coding RNAs. MiR-14 was one of the non-coding RNAs that played an important role in egg-laying [11,14]. Our study demonstrated that the expression of miR-14 and EcR was significantly associated with the number of eggs laid in honeybee queens. Once the results are confirmed in other populations, this study, in combination with traditional selection methods, can be used for marker-assisted selection in honeybee breeding programs. However, there may be other genes or loci interacting with EcR or miR-14. Specific mechanisms are needed to carry out further studies.

## 5. Conclusions

In summary, we found that inhibition of miR-14 and overexpression of EcR enhanced the number of eggs laid by queens. Moreover, we determined that miR-14 directly targets the mRNA of EcR. These findings suggest that miR-14, by targeting EcR, plays an important role in regulating honeybee egg-laying.

## Figures and Tables

**Table 1:** Primer sequences used for qRT-PCR validation of miR-14 and EcR.

Primer	5′ to 3′
miR-14-F	GCGCTCAGTCTTTTTCTCT
U6	CTTGCTTCGGCAGAACATAT
EcR-F	GCCTCCGGTTACCACTACAA
EcR-R	CTCGCAATTGTTCCCGTATT
β-actin-F	CTGCTGCATCATCCTCAAGC
β-actin-R	GAAAAGAGCCTCGGGACAAC

## Supplementary Materials

The following are available online at https://www.mdpi.com/article/10.3390/insects12040351/s1, Figure S1 Counting the number of eggs per day using Population Measurment Liebefeld method. Table S1: Sequence of EcR 3′UTR.
