# Supplementary material for: The microRNA miR-14 Regulates Egg-Laying by Targeting EcR in Honeybees (Apis mellifera)
Source: Insects. 2021 Apr 14;12(4):351. doi: 10.3390/insects12040351 (PMC8071020; doi:10.3390/insects12040351)

Table S1 Sequence of EcR 3’UTR

Gene	Sequence
EcR 3’UTR	5’ ttcccc ggtggccgat ccccgtcggc tgatagtgtt gtttcatcgc gagtccgcgt cgcgcccgga acgttcaagc tgaacaccga atggagctgc ggaacgctgc gcgccacgcg gagaacctcc tgtactagtg cgccaccacc accaccacca ccaccaccat gccactgcct ggcccgatta gtagacacag gtgacgtcta ccgaagaagc atcgaggaat gatcgattcg acttcatcac accttcttct ctccgcactt cacccggctt cgtgaagctg tgctggaaac gggggggggg gcagctttcg acagtgcttt acgaaaacgc ttagaaacgc tgtggaggat acgataataa tattttacgc gaagtcagga ggaaggagga aggaggaagg aggaaaggca gtggcgaggc acccaggctg tgatacgtag agacagtaca gagactgtaa taaggagcta cctacctaca gttacgttat tattactata ttctctacct ggctacctac ctacctacct acctacctac ctacctaccta cctacctac ctacctacc 3’

Fig. S1 Counting the number of eggs per day using Population Measurment Liebefeld method.

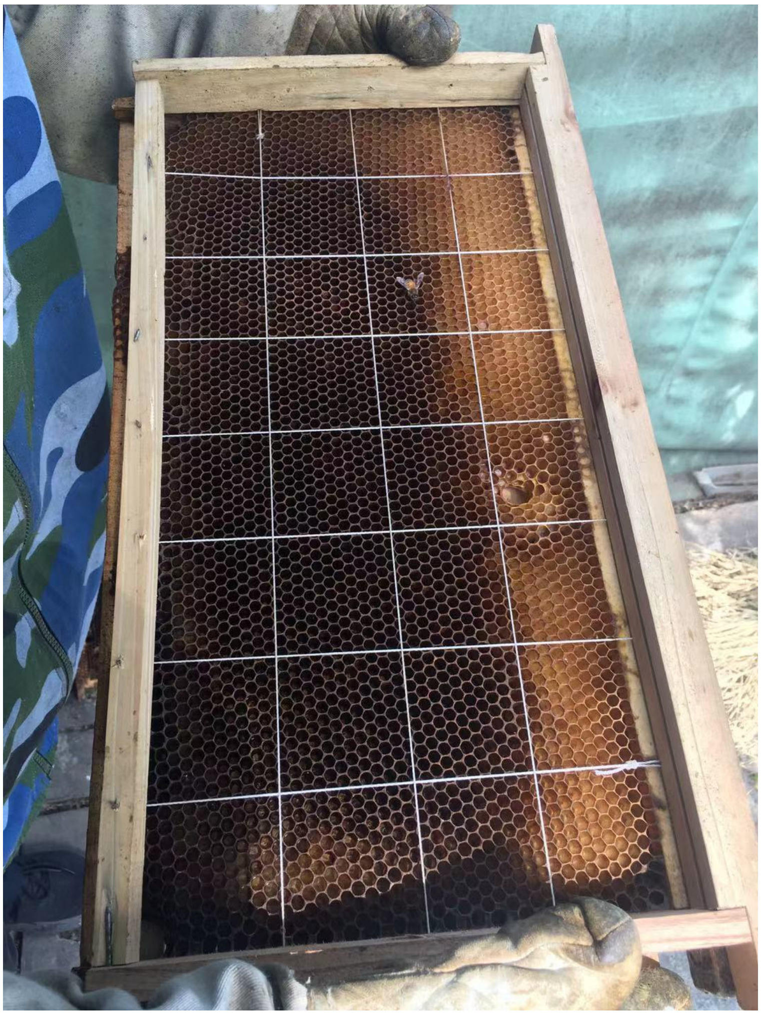

Supplement: Supplementary file 1 [file insects-12-00351-s001.pdf]
